# Understanding the Metabolic Profile of Macrophages During the Regenerative Process in Zebrafish

**DOI:** 10.3389/fphys.2019.00617

**Published:** 2019-05-24

**Authors:** Lais Cavalieri Paredes, Niels Olsen Saraiva Camara, Tarcio Teodoro Braga

**Affiliations:** ^1^Department of Pathology, Federal University of Parana, Curitiba, Brazil; ^2^Department of Immunology, Institute of Biomedical Sciences IV, University of São Paulo, São Paulo, Brazil; ^3^Nephrology Division, Federal University of São Paulo, São Paulo, Brazil; ^4^Renal Pathophysiology Laboratory, Faculty of Medicine, University of São Paulo, São Paulo, Brazil

**Keywords:** regeneration, macrophage, redox balance, ROS production, zebrafish

## Abstract

In contrast to mammals, lower vertebrates, including zebrafish (*Danio rerio*), have the ability to regenerate damaged or lost tissues, such as the caudal fin, which makes them an ideal model for tissue and organ regeneration studies. Since several diseases involve the process of transition between fibrosis and tissue regeneration, it is necessary to attain a better understanding of these processes. It is known that the cells of the immune system, especially macrophages, play essential roles in regeneration by participating in the removal of cellular debris, release of pro- and anti-inflammatory factors, remodeling of components of the extracellular matrix and alteration of oxidative patterns during proliferation and angiogenesis. Immune cells undergo phenotypical and functional alterations throughout the healing process due to growth factors and cytokines that are produced in the tissue microenvironment. However, some aspects of the molecular mechanisms through which macrophages orchestrate the formation and regeneration of the blastema remain unclear. In the present review, we outline how macrophages orchestrate the regenerative process in zebrafish and give special attention to the redox balance in the context of tail regeneration.

## Zebrafish and Tail Fin Regeneration

Zebrafish is a teleost fish that has been studied and increasingly utilized over the last three decades as an experimental animal model in several areas of science (Renninger et al., 2011; Weyand and Shavit, 2014). The species *D. rerio* belongs to the Animalia kingdom, Chordata phylum, Actinopterygii class (Teleostei infraclass), Cypriniformes order and Cyprinidae family. It is a species of small size that measures approximately 3 cm in adulthood and lives in freshwater rivers and lakes, and it is native to the Himalayan region. Males and females can be anatomically distinguished in a relatively simple way; the males have a thinner body, more prominent black bands throughout the body (which is the origin of the name zebrafish), and a notable golden coloration on the belly and fins. Females, on the other hand, tend to have a more prominent abdominal region, especially during the period of oviposition, and have less gold coloration on the body (Wixon, 2000).

Several factors contribute to the suitability of zebrafish as a research model (Hoo et al., 2016). First, zebrafish are easily accessible, have low maintenance costs, are very simple to care for compared to other animal models and are maintained very easily in a laboratory environment due to their small size. Zebrafish are oviparous fish that exhibit external fertilization and very fast embryonic development that lasts from 2 to 4 days. The embryos of the species are translucent, which facilitates the visualization of structures under a simple optical microscope (Darrow and Harris, 2004; Zhao et al., 2011). In addition, comparison with the human reference genome shows that approximately 70% of human genes have at least one clear zebrafish ortholog, which improves confidence in using it as a model and indicates the potential implications of research results (Howe et al., 2013). The caudal fins of zebrafish comprise 16 to 18 bony rays, which extend along the tail and are separated by interray tissue. These bones are segmented and covered by an epidermis, where blood vessels, nerves, pigmented cells and fibroblasts are observed. The fins are easily surgically removed and exhibit fast and healthy regeneration (Wixon, 2000; Wehner and Weidinger, 2015; Sehring et al., 2016).

An understanding of the processes of fibrosis and tissue regeneration is extremely important for the elucidation of several diseases and traumas that originate from impaired regeneration (Wynn and Ramalingam, 2012). Whenever an injury occurs, there is an exchange of regenerative and fibrotic states in the tissues that depends on the affected tissue and the regenerative capacity of that species (Julier et al., 2017). All animals have developed strategies to respond to diseases and injuries, but these strategies differ greatly between species, such as mammals and amphibians/fish (Fumagalli et al., 2018). The latter are capable of completely regenerating the heart, retina, limbs, and other organs (Gemberling et al., 2013; Wan and Goldman, 2016). There are three distinct regeneration processes in vertebrates; of these, epimorphic regeneration occurs in zebrafish. The other processes are tissue regeneration, which is characterized by the predominant repair of a single cell type, and compensatory growth, which is exemplified by the regeneration of the liver after partial hepatectomy in humans (Iismaa et al., 2018).

**FIGURE 1:**
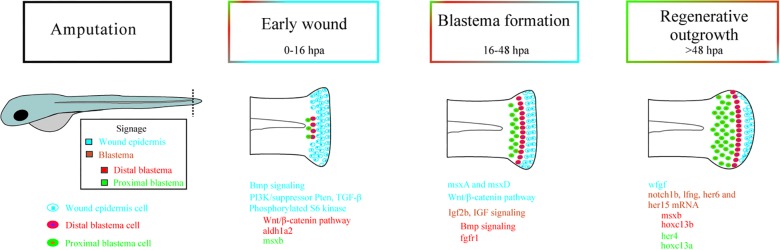
Signaling during epimorphic regeneration in zebrafish followed by tail amputation. Cellular signaling processes involved in three different phases of caudal fin regeneration: early wound, blastema formation, and regenerative outgrowth. The signaling is separated into different regions. The wound epidermis, represented in blue, is characterized by signaling via BMP, PI3K/suppressor Pten, TGF-β, and PS6K in the early wound (0–16 h postamputation). During blastema formation (16–48 hpa), the wound epidermis is characterized by both msxA and msxD expression and the Wnt/β-catenin pathway. Finally, fgf is expressed during regenerative outgrowth (>48 hpa) in the wound epidermis. The signaling that arises from the distal blastema is represented in red, which shows that the Wnt/β-catenin pathway and the aldh1a2 gene are expressed in early wounds. BMP signaling and fgfr1 are expressed during blastema formation and msxB and hoxc13b during regenerative outgrowth. The green color represents the proximal blastema, in which msxB is expressed in the early wound and hoxc13a, her4, and msxB are expressed during regenerative outgrowth. In orange, the signaling processes that happen throughout the blastema, the Igf2b and IGF signaling that occur during blastema formation and the expression of notch1b, lfng, her6, and her15 mRNA during regenerative outgrowth are shown. BMP: Bone morphogenetic protein; PI3K/suppressor Pten: Phosphoinositide 3-kinase/suppressor phosphatase and tensin homolog; TGF-β: Transforming growth factor beta; PS6K: Phosphorylated S6 kinase; msxA: Homeobox protein MSH-A; msxD: Homeobox protein MSH-D; fgf: Fibroblast growth factor; aldh1a2: Aldehyde dehydrogenase 1 family member A2; msxB: Homeobox protein MSH-B; hoxc13b: Homeobox C13b; hoxc13a: Homeobox C13a; her4: Human epidermal growth factor receptor 4; Igf2b: Insulin-like growth factor 2b; IGF: Insulin-like growth factor; Notch1b: Notch homolog 1b; lfng: LFNG O-Fucosylpeptide 3-Beta-N-Acetylglucosaminyltransferase.

Epimorphic regeneration in zebrafish occurs via the formation of a blastema that is characterized by a population of progenitor cells capable of interacting with epithelial cells and is necessary for the reconstruction of the injured area (Pfefferli and Jaźwińska, 2015). The first step in this process is the rapid formation, via migration, of a layer of cells in the epidermis in the lesioned region that is referred to as the wound epidermis (WE), which later specializes and develops a different gene expression profile from that found in normal epidermal tissue. Concomitantly, angiogenesis, which requires vascular endothelial growth factor (VEGF) signaling, occurs (Stoick-Cooper et al., 2007). The second step is the formation of the blastema itself, which is a proliferative mass of undifferentiated precursor cells that seems to be induced by signals produced in the WE. Between two and four days postinjury in adult zebrafish, the tail begins to recover its growth due to an increase in the cell cycle velocity that allows it to complete in 1 h instead of the approximately 6 h that is normally required (Nechiporuk and Keating, 2002; Stoick-Cooper et al., 2007). Finally, the blastema cells begin to differentiate into other cell types, such as scleroblasts, which secrete a matrix to form new bones (Stoick-Cooper et al., 2007). A schematic view of the regenerative process is shown in Figure 1 and is described in better detail in Table 1.

**Table 1:** Signaling processes triggered during regeneration.

Expression of	Region	Time	References
*wfgf*	*Wound epidermis*	Regenerative outgrowth (48 hpa)	Poss et al., 2000
*fgfrl*	Mesenchymal cells underlying we/Distal blastema tissue	Blastema formation (24 hpa)/regenerative outgrowth (48 hpa)	Poss et al., 2000
*msxb*	Proliferating cells (proximal blastema)/most distal blastema (MDB)	Early blastema formation (16 h)/regenerative outgrowth	Nechiporuk and Keating, 2002
Notch target gene locus *her4*	Proximal blastema	Regenerative outgrowth	Grotek et al., 2013
*aldh1a2 (raldh2 – enzyme that catalyzes retinoic acid synthesis*	most distal blastema (MDB)	Regenerative outgrowth	Mathew et al., 2009; Blum and Begemann, 2012
*hoxcl3b*	Distal blastema	84 hpa	Thummel et al., 2007
*hoxcl3a*	Proximal blastema	60 hpa	Thummel et al., 2007
*msxA* and *msxD*	*Wound epidermis*	>30 hpa	Akimenko et al., 1995
Phosphorylated S6 kinase (p-S6K) mTORC1 signaling	Intra-ray and *wound epidermis*/*Wound epidermis* and blastema	6–18 hpa/>24 hpa	Hirose et al., 2014
*notch1b*, *lfng*, *her6*, and *her15* mRNA	Blastema	72 hpa	Münch et al., 2013
Wnt/p-catenin	Non-proliferative distal blastema	Soon after amputation and maintained throughout the course of regeneration	Wehner et al., 2014
*Shh, lef1* and Hh signaling	Proximal subregion of the basal epidermal layer		Wehner and Weidinger, 2015
*Igf2b*, IGF signaling	Blastema	24–36 hpa	Chablais and Jazwinska, 2010

It has been demonstrated that the proliferation of the blastema and WE modeling in adult zebrafish is governed by the Wnt/β-catenin signaling pathway, especially during the direct regulation of cellular proliferation and the maintenance of stem cells and other specific progenitor cells (Kawakami et al., 2006). However, instead of being active in the proliferating blastema, it is known that Wnt/β-catenin signaling occurs in the non-proliferative region, where this signaling contributes to the proliferation of the proximal region. In addition to being responsible for the regulation of the WE and the proliferation of the blastema, the Wnt/β-catenin pathway also controls the maturation of osteoblasts via secondary signals (Baron and Rawadi, 2007). Some experiments suggest that this pathway acts through the signaling of fibroblast growth factor (FGF) and bone morphogenetic protein (BMP) to control the pattern of epidermal gene expression through retinoic acid signaling and Hedgehog (Hh) signaling, which has an important role in regulating the development of several embryonic organs (Wehner et al., 2014). In addition to these pathways, there are many other pathways described in the literature that are necessary for caudal fin regeneration in zebrafish (Poss et al., 2000; Blum and Begemann, 2012; Wehner and Weidinger, 2015), including the Notch, insulin-like growth factor (IGF) (Chablais and Jazwinska, 2010; Huang Y. et al., 2013; Saera-Vila et al., 2018), activin (Jaźwińska et al., 2007), mechanistic target of rapamycin complex 1 (mTORC1) (Hirose et al., 2014), calcineurin (Wixon, 2000), and fibroblast growth factor receptor (*fgfr*) (Saera-Vila et al., 2016) signaling pathways as well as the signaling pathways that involve the homeobox class genes, including those encoding the homeobox msx and hox proteins (Akimenko et al., 1995; Thummel et al., 2007). A quantitative proteomic study performed during epimorphic tail regeneration demonstrated differential protein regulation in the lesion and in control tissue (Saxena et al., 2012). Most of these proteins were related to the maintenance of cellular structure and architecture, while others were associated with the immune system and the cytoskeletal remodeling pathway, including keratin and its ten isoforms, cofilin-2, and annexin A1 (Saxena et al., 2012).

It is known that the blastema is responsible for supplying all cell types that make up the caudal fin and therefore restoring the structural pattern of the tail after the lesion. The progenitor cells of the blastema remain undifferentiated due to the activation of the Notch pathway (Grotek et al., 2013). This pathway, which is initially activated during the formation of the blastema, remains active throughout the regenerative process (Grotek et al., 2013). It has been shown that the chemical or genetic inhibition of the Notch pathway impairs regeneration by decreasing cell proliferation and decreasing the expression of target genes, such as *her6* and *her15*, in the blastema (Münch et al., 2013). In a complementary manner, the overexpression of an active form of the Notch 1 receptor (N1ICD) leads to the increased proliferation and expansion of undifferentiated cells expressing markers that include *msxe*, *msxb* and the negative regulator of proliferation *aldh1a2*. Thus, the Notch signaling pathway maintains the cells of the blastema in a plastic, undifferentiated and proliferative state, which is essential for the complete regeneration of the caudal fin (Münch et al., 2013).

Moreover, the increased expression of hyaluronic acid synthase 3 (*has3*) was identified during caudal fin regeneration in zebrafish larvae (Ouyang et al., 2017). *has3* expression was demonstrated 6 h after amputation in fish 2 days after fertilization and reached maximum levels within the first day. The initiation and maintenance of *has3* transcription is restricted to the WE and requires several active signaling pathways, which are initiated by FGF, phosphoinositide 3-kinase (PI3K) and tumor growth factor beta (TGF-β). The inhibition of hyaluronic acid (HA) synthesis using a small molecule, 4-methylumbelliferone (4-MU), reduces tail regeneration in zebrafish larvae by preventing lesion-induced cell proliferation (Ouyang et al., 2017).

In addition to the formation of the epidermal layer and the blastema, regeneration depends on tissue morphogenesis (Jaźwińska and Sallin, 2016). One study conducted by the Jazwinska group revealed that the activity of bone morphogenetic protein (BMP) is responsible for WE maintenance and osteoblast and blood vessel formation (Thorimbert et al., 2015). Moreover, it has been observed that the phosphatase and tumor suppressor Pten, which is an antagonist of phosphoinositide-3-kinase (PI3K) signaling, is required for caudal fin fold regeneration in zebrafish larvae (Hale et al., 2017). A recent study found that homozygous knockout mutant zebrafish embryos lacking functional Pten (ptena^−/−^ptenb^−/−^) were not capable of regenerating their caudal fin folds. These organisms showed decreased proliferation and enhanced apoptotic activity. The reinduction of Pten expression or pharmacological inhibition of PI3K, in turn, rescued the regenerative process (Hale et al., 2017). In addition to proliferative cells from the blastema, several studies have shown that macrophages play an important role in regenerative processes through protein synthesis, angiogenesis, the production and release of cytokines such as VEGF and interleukin 1 (IL-1), and the promotion of cell proliferation (Koh and DiPietro, 2011; Wynn and Vannella, 2016; Godwin et al., 2017; Abnave and Ghigo, 2018).

### Zebrafish and Heart Regeneration

In addition to some amphibians, teleost fish, such as zebrafish, have remarkable regenerative capacity. After a 20% ventricular resection, the zebrafish can fully regenerate its heart within 2 months (Poss et al., 2002; Chablais et al., 2011). Some injury models have been developed that induce myocardial lesions, mainly by using methods of resection, cryoinjury and genetic ablation, in zebrafish (Curado et al., 2007, 2008; Chablais et al., 2011; Kikuchi et al., 2011). A critical goal of regenerative biology is the identification of the cellular origins of regenerating tissues. Two different studies examined the contribution of cardiomyocytes (CMs) to the regeneration of the heart in zebrafish by using inducible genetic fate-mapping techniques, such as Cre-lox (Jopling et al., 2010; Kikuchi et al., 2010). It was shown that regenerated heart muscle cells are derived from the proliferation of differentiated CM (Jopling et al., 2010). In addition, it has been demonstrated that atrial CM can transdifferentiate into ventricular CM to contribute to cardiac ventricular regeneration in zebrafish. The same authors have observed that Notch signaling becomes activated in the atrial endocardium following ablation of CM in the ventricular area through genetic manipulation. Moreover, the inhibition of this signaling pathway was shown to block atrial-to-ventricular transdifferentiation and cardiac regeneration (Zhang et al., 2013). The significance of the Notch signaling pathway in heart regeneration was demonstrated in another study, in which manipulation of Notch and Serpine1, both key endocardial factors, interfered with inflammatory responses and CM proliferation and dedifferentiation. Upon Notch inhibition, an elevated number of plastin^+^ and mpeg1^+^ macrophages was found to be associated with wound endocardial cells, and increased expression of ECM-degrading proteases and inflammatory and regulatory markers was observed. These results suggest that Notch is responsible for restricting inflammatory responses at the site of injury (Münch et al., 2017).

Several pathways have also been shown to be required for both fin and heart regeneration (Sehring et al., 2016), such as those regulating FGF (Poss et al., 2000; Lepilina et al., 2006; Whitehead et al., 2006), Hh (Quint et al., 2002; Zhang et al., 2012; Choi et al., 2013; Wang et al., 2015), BMP (Quint et al., 2002; Smith et al., 2006; Stewart et al., 2014; Wu et al., 2016), RA (Kikuchi et al., 2011; Blum and Begemann, 2015a,b), IGF (Chablais and Jazwinska, 2010; Choi et al., 2013; Huang Y. et al., 2013), Cxcl12/Cxcr4 (Itou et al., 2012; Xu et al., 2014) and ROS (Gauron et al., 2013; Han et al., 2014); some of these pathways have also been shown to be important for heart regeneration, including those regulating platelet-derived growth factor (PDGF) (Kim et al., 2010), TGFβ (Chablais and Jazwinska, 2012; Choi et al., 2013), Janus kinase/signal transducers and activators of transcription (JAK/STAT) (Fang et al., 2013), neuregulin (Gemberling et al., 2015), nuclear factor-κB (NF-κB) (Karra et al., 2015) and MAPK/ERK (Liu and Zhong, 2017). A recent study demonstrated that a significant reduction in CM proliferation in resected hearts compared to that in control hearts was mediated through suppression of MEK1/2, which is a mitogen-activated kinase involved in the MAPK/ERK signaling pathway; this implies that MAPK/ERK signaling is required during zebrafish cardiac regeneration (Liu and Zhong, 2017). Additionally, the usage of a dominant-negative form of MEK1 led to the inhibition of cardiac regeneration and an increase in cardiac fibrosis with the absence of endothelial cells in the wound area, which suggested that MAPK/ERK signaling may regulate cardiac regeneration by influencing angiogenesis or the migration of endothelial cells (Liu and Zhong, 2017).

Through the generation of a dynamic coexpression network of heart regeneration molecules in zebrafish, it has been shown that regeneration steps are mediated by modules of transcriptionally coordinated genes and by genes acting as network hubs. As early as 4 h postinjury (hpi), genes involved in apoptosis, angiogenesis and cell migration have been identified. Energy metabolism, DNA replication and amino acid biosynthesis are processes that have been shown to be the most impacted at 1-day postinjury (1 dpi), which might reflect enhanced cell proliferation. At the same time, point changes are also observed in genes related to proteolytic activities; later, at 3 dpi, the ECM is involved. Processes involved in cell adhesion are mainly detected at later times (Rodius et al., 2016).

Tissue-resident macrophages, the most abundant immune cell population in the heart, play a crucial role in cardiac regeneration in several animal models (Aurora et al., 2014; Pinto et al., 2014; Lai et al., 2017). These cells display a gene expression profile similar to that of anti-inflammatory M2 macrophages; in adult mice, they are replaced with or outnumbered by monocyte-derived macrophages, which are prominent pro-inflammatory cells, after CM ablation (Lavine et al., 2014). In contrast, after myocardial injury in neonatal mice, tissue-resident macrophages expand and assist cardiac recovery by promoting angiogenesis and CM proliferation (Lavine et al., 2014). In another model, the depletion of phagocytes via clodronate liposomes altered angiogenesis and regeneration (Aurora et al., 2014). Correspondingly, the depletion of macrophages by clodronate liposomes prior to cryoinjury in zebrafish led to defects in revascularization and CM proliferation (Lai et al., 2017, 2019).

By using a non-steroidal anti-inflammatory drug, ibuprofen, Huang and colleagues demonstrated that zebrafish hearts, upon ventricular resection, showed a significant reduction in their regenerative capacity, and substantial amounts of residual scar tissue and cellular debris were observed within the wound at 1 month postinjury. Using a similar approach with betamethasone, a glucocorticoid, it was shown that this treatment impaired immune system processes, such as macrophage activation, via gene ontology analysis (Huang W.C. et al., 2013).

## Macrophages

Macrophages are immune cells of myeloid origin that are strategically positioned in tissues that play roles in the defense of the body and in inflammatory processes and that are important for homeostasis and tissue development (Gordon and Martinez-Pomares, 2017). Macrophages can be derived from monocytes and the yolk sac. They are present in almost all tissues and exhibit distinct phenotypes and gene expression patterns depending on their location (Haldar and Murphy, 2014). Since these cells exhibit several levels of heterogeneity and plasticity, they are key factors in several pathologies and contribute to the development of diseases, such as atherosclerosis, or rescue of function, such as occurs during the regeneration of some organisms with high regenerative capacity (Varol et al., 2015).

Macrophages, in general, were originally classified into two phenotypes: M1, which have pro-inflammatory characteristics, and M2, which have immunoregulatory characteristics (Martinez and Gordon, 2014). Due to restrictions in these classifications, M2 macrophages were later classified according to the role they play in cells, such as in wound healing (M2a), inflammation-related and immunocomplex-induced processes (M2b), and regulatory processes (M2c) (Rőszer, 2015). A common macrophage nomenclature, however, has been proposed that depends on the source of macrophages, which is based on the consensus collection of markers or the differentiating stimuli from activators (Murray et al., 2014). M1 cells are intimately related to Th1 CD4^+^ T cells, and M2 cells are intimately related to Th2 CD4^+^ T cells. M1 macrophages are differentiated by stimulation with IL-12, LPS or IFN-γ or in response to acute inflammation, whereas M2 cells can be differentiated by stimulation with IL-4 or IL-13 (Tacke and Zimmermann, 2014). The main functions of M1 macrophages are the elimination of bacterial agents, antiviral activity and the release of proinflammatory cytokines such as TNF-α, IL-1β and reactive oxygen species (ROS). M2 macrophages, on the other hand, promote defense against parasites and may be involved in tissue repair as well as the secretion of immunomodulatory mediators such as IL-10, TGF-β, IL-4 and IL-13. Schematic representations of macrophage polarization are better represented in detail in Braga et al. (2015) and Rőszer et al. (Rőszer, 2015). Depending on the pathological scenario, macrophages may simultaneously express markers corresponding to both phenotypes, which demonstrates that macrophages are plastic cells that travel within the spectrum between M1 and M2 rather than cells with stable characteristics (Cao et al., 2015).

During infections and injuries, macrophages are activated by pathogen-associated molecular patterns (PAMPs) and damage-associated molecular patterns (DAMPs), and these cells start producing and secreting antimicrobial mediators, cytokines and chemokines (Jounai et al., 2012). An exacerbated reaction may lead to a fibrotic process, culminating in scar formation and, as a consequence, a loss of function. In these situations, it is crucial that the macrophage population is converted to a phenotype with anti-inflammatory or regulatory characteristics so that collateral damage to the tissue is attenuated. Several mediators convert macrophages, including IL-4, IL-13, the Fcγ receptor and the TLR signaling pathway (Elliott and Hamilton, 2011). Regulatory cells synthesize substances such as arginase 1 (ARG1), IL-10, MMP13, CD200, maresins, RELMα, IKKα and PD-L2, all of which contribute to a reduction in inflammatory responses and to the resolution of fibrosis. Furthermore, they produce CSF1, IGF1 and VEGF, which promote wound healing (Serhan et al., 2009; Koning et al., 2010). These signals are closely related to the recovery phase of fibrosis, during which they induce the degradation of the extracellular matrix and phagocytosis of apoptotic myofibroblasts and cellular debris as well as the regulation of immune responses (Wynn et al., 2013).

### Macrophages and Regeneration

Macrophages contribute in different ways to the maintenance and recovery of tissue homeostasis. They are present during the process of tissue repair and regeneration, and their beneficial effects are attributed to the tropic factors they release in the cellular environment (Van Gassen et al., 2015). By using macrophage depletion approaches, it has been shown that macrophages are critically involved in tissue repair after skin, liver, kidney and muscle injury (Chazaud, 2014). Additionally, it has been reported that myeloid-defective zebrafish mutants exhibit apoptosis in regenerative cells during regeneration of the caudal fin; it was discovered that this phenotype is induced by the prolonged expression of interleukin 1 beta (IL-1β). The main source of this cytokine is myeloid cells, but epithelial cells also express it in response to tissue injury, which contributes to the initiation of inflammation (Hasegawa et al., 2017).

It is known that macrophages have different transcriptional profiles that are important in maintaining metabolic homeostasis (Jantsch et al., 2014). After an injury, resident macrophages and newly arriving monocytes start to exhibit highly flexible and shifting programs of gene expression and critical regulatory responses throughout all phases of repair and fibrosis (Mescher, 2017). Macrophages are induced by cytokines to become important sources of cytokines, matrix metalloproteinases (MMP), chemokines and other factors that are involved in many steps of the inflammatory response (Mescher, 2017).

There is an important difference between the metabolism of inflammatory and anti-inflammatory macrophages. Inflammatory macrophages possess increased aerobic glycolytic pathway signaling and an active pentose pathway, whereas the anti-inflammatory phenotype is characterized by the oxidative metabolism of glucose, which is represented by the oxidation of fatty acids and is possibly responsible for supporting its longer lasting functions, such as tissue remodeling and repair (Langston et al., 2017). Another distinction between the phenotypes is related to iron storage, as inflammatory macrophages express a large amount of ferritin whereas anti-inflammatory macrophages express ferroportin, the main exporter of iron. In addition, M1 polarization induces the use of arginine by nitric oxide synthase 2 (NOS2) to produce nitric oxide for the elimination of exogenous agents, whereas polarized M2 macrophages convert arginine to polyamines via ARG1, which is involved in the synthesis of collagen and in cell proliferation (Chazaud, 2014; Das et al., 2015; Van Gassen et al., 2015).

In vascularized tissues, injury induces an inflammatory response characterized by the presence of M1 macrophages, which are responsible for the restriction of the injured area and for the cleansing of cellular debris. The second phase is characterized by tissue repair or regeneration, which can occur if the parenchyma can recover its functioning. This process involves the presence of M2 macrophages; however, there is a lack of data regarding the direct role of M2 macrophages in tissue regeneration (Chazaud, 2014). Macrophages are found in greater numbers in pathological conditions. In the context of injury or infection, DAMPs and PAMPs increase the presence of pro-inflammatory macrophages that contribute to increased tissue injury, inflammation and consequent formation of fibrosis (Land, 2015). On the other hand, cells undergoing apoptosis and anti-inflammatory factors induce macrophages with an anti-inflammatory profile, which appear to have regenerative capacity and tissue repair activity (Cao et al., 2015).

Macrophages that infiltrate injured regions are known as wound macrophages. Several studies suggest that the polarization of these cells is extremely dynamic and dependent on the environment of the lesion (Daley et al., 2010; Brancato and Albina, 2011). One study reported that infiltrating macrophages have characteristics typical of both classically and alternatively activated macrophages and are predominant in the production of proinflammatory cytokines such as TNF-α and IL-6 on day 1 postinjury and TGF-β on day 7. In sterile lesions, the induction of inflammation is very similar to that in lesions caused by pathogens due to the induction of activation by endogenous markers of damage, such as amphoterin (HMGB1), iron, histones or ATP (Lech and Anders, 2013; Das et al., 2015).

Additionally, during caudal fin regeneration, an early and transient accumulation of pro-inflammatory macrophages is simultaneously observed along with the accumulation of anti-inflammatory macrophages, and the latter remain associated with the fin until the end of regeneration (Hasegawa et al., 2017). The genetic and chemical depletion of macrophages indicated that macrophages that are recruited early and that express TNF-*α* are critical for blastema formation. By combining parabiosis and morpholino knockdown strategies, a study reported that the TNF-*α*/TNFR1 signaling pathway is required for fin regeneration and revealed that TNFR1 has a necessary and direct role in blastema cell activation, which suggests that the balance of macrophage subtypes provides an accurate TNF-*α* signal to prime regeneration in zebrafish (Nguyen-Chi et al., 2017). Macrophages also have the ability to transdifferentiate into endothelial cells and endothelial progenitor cells in the presence of pleiotrophin (PTN) (Sharifi et al., 2006) or through VEGF overexpression, both demonstrated *in vitro* (Yan et al., 2017). The generation of endothelial cells through macrophage transdifferentiation is very important and represents an aspect of plasticity that could be used to stimulate the revascularization of injured tissue in ischemic lesions (Das et al., 2015).

In contrast to zebrafish, another fresh water teleost, medaka (*Oryzias latipes*), has been noted to lack CMs proliferation and revascularization after cardiac injury, which results in excessive fibrotic responses and unresolved scars (Ito et al., 2014). Following heart cryoinjury, a comparative transcriptomic analysis was performed, and it was observed that acute immune responses appeared to be different in these two teleost fishes. Using sample level enrichment analysis (SLEA), it was shown that zebrafish presented a stronger activation of genes related to the complement system, B cells, T cells, macrophages and phagocytosis, while medaka showed stronger activation of genes involved in neutrophil and monocyte activation. Zebrafish also demonstrated a stronger and prolonged activation of cell proliferation and angiogenesis-related genes (Lai et al., 2017).

In addition, comparisons between zebrafish and medaka cardiac regeneration have indicated that macrophage recruitment, angiogenesis and cell proliferation in medaka are delayed and reduced. Moreover, after the injection of poly I:C, which is a TLR3 and NLRC4 agonist, the medaka heart showed reduced scar tissue at 1 month postinjury when compared to the non-stimulated control heart. These data indicate that harnessing the acute immune response through TLR signaling activation can induce heart regeneration in a species not fully capable of regenerating its heart by promoting macrophage recruitment, neovascularization, neutrophil clearance, CM proliferation and scar resolution (Lai et al., 2017).

Recently, it has been demonstrated that, as early as 1-day postcryoinjury, an injured area in a zebrafish heart shows an increase in matrix metalloproteinase (MMP) enzymatic activity and elevated expression of *mmp9* and *mmp13* (Xu et al., 2018). The specific inhibition of both MMP-9 and MPP-13 resulted in impaired regeneration and leukocyte recruitment. Through the use of a broad-spectrum MMP inhibitor and subsequent chemokine rescue using recombinant CXCL8 and CCL2, the restoration of macrophage recruitment and cardiac regenerative capability in these fishes was observed, suggesting that MMPs might play a key role in the inflammatory phase of heart regeneration in zebrafish by promoting leucocyte recruitment via the activation of chemokines (Xu et al., 2018).

Furthermore, in an adult zebrafish caudal fin model, the recruitment of neutrophils and macrophages has been well established by live cell imaging *in vivo* (Ellett et al., 2011). Both embryonic and larval caudal fins have a high regenerative capacity (Johnson and Weston, 1995; Kawakami et al., 2004). A study in an embryonic model that used interferon regulatory factor 8 (irf8)-morpholino macrophage depletion suggested that macrophages are required for normal postinjury growth and proliferation (Jin et al., 2012; Keightley et al., 2014). Additionally, recent studies have shown that immune cells are crucial for the regulation of pro-inflammatory and pro-regenerative signals that shift the injury microenvironment toward regeneration (Cheng et al., 2017; Horckmans et al., 2017; Julier et al., 2017; Rodriguez and Yin, 2019). The role of macrophages during regeneration has been highlighted through the demonstration of their function in neutrophil clearance by phagocytosis, which prevents a prolonging of the inflammatory phase in the injured area (Lai et al., 2017). In addition, these highly plastic cells are responsible for the mediation of cardiac fibrosis via their capacity to directly release matrix proteins. Furthermore, they promote the proliferation and activation of fibroblasts by the release of stimulatory cytokines (Pinto et al., 2014). Subsequently, to allow scar regression, macrophages inactivate fibroblasts, which shuts off the pro-fibrotic response (Godwin et al., 2017), and begin to release MMPs to break down the extracellular matrix that was previously formed (Leor et al., 2016).

New evidence has indicated that zebrafish macrophages can play different roles during immune responses involved in tissue restoration after infection or damage. A recent study distinguished peripheral from hematopoietic tissue-resident macrophages during tail fin regeneration, and distinct differences in migratory behavior were observed between these two macrophage populations. Additionally, the specific role of peripheral tissue-resident macrophages in tail fin regeneration has been reported via the downregulation of inflammatory mediators such as interleukin-1b (*il1b*) and the depletion of ROS at the damage site (Morales and Allende, 2019).

## Metabolism and Regeneration

Recently, much attention has been given to the role of metabolism, especially in cells of the immune system. For instance, macrophages maintain pathways such those involved in glycolysis, glutaminolysis, amino acid transport and fatty acid oxidation; depending on their state of activation and differentiation, this process directly impacts their effector functions (Buck et al., 2015, 2017). Cell metabolism is involved in various enzyme-orchestrated chemical reactions with the purpose of generating energy for cells to perform their functions. Cellular metabolism can be divided into catabolism and anabolism. Catabolism is the process by which larger molecules are metabolized into smaller ones to generate energy, while anabolism is the process by which smaller molecules are used to generate larger molecules, which is important for cellular structure.

Cells in states of activation and proliferation use glycolysis as the primary source of energy, i.e., the generation of ATP. Normally, glycolysis occurs in the cytoplasm of the cell and generates the pyruvate molecule as the final product, which is then transformed into acetyl-CoA; in the mitochondria, this is metabolized in the TCA cycle, which generates several intermediate products that will be used in the respiratory chain to generate ATP. However, proliferating immune cells and, to a lesser extent, classically activated macrophages use anaerobic glycolysis, which is detrimental to oxidative phosphorylation, as a source of energy; however, they do so in the presence of oxygen (WARBURG, 1956). Instead of the pyruvate that is generated in glycolysis entering the TCA cycle to be metabolized and generate products for oxidative phosphorylation, it is transformed into lactate. This phenomenon is described as the Warburg effect, and it is believed that, although it produces little ATP, cells rely on it for the production of intermediates such as NAD and FAD that are necessary for the execution of several cellular processes that promote cell growth and proliferation.

It is now known that resting cells and tissue resident macrophages (M2) use fatty acid metabolism for their survival, while M1 macrophages are glycolysis-dependent (Everts et al., 2014; Blagih et al., 2015) and in some cases, use glutaminolysis for activation (Huang et al., 2014; Blagih et al., 2015). Recent studies indicate that this dichotomy is increasingly difficult to establish (Gerriets et al., 2016; Phan et al., 2016). Several intermediates of cellular metabolism, such as NADH and FADH, serve to promote the synthesis of ATP in the mitochondria and ultimately aid in cell survival. On the other hand, activation of these cells can be harmful; allowing persistent activation of these cells may impact their migration (Huang et al., 2014; Gerriets et al., 2016). The impact of these metabolic pathways on the function of immune cells is directly related to the development of diseases such as fibrotic diseases.

The mammalian rapamycin target protein (mTOR) is part of a signaling pathway that participates in various cellular functions, such as metabolism, growth, survival, aging and memory (Wullschleger et al., 2006). mTOR is an evolutionarily conserved serine-threonine kinase with a molecular weight of 290 kDa. Its name originates from its inhibitor rapamycin (RAPA), which is also known as Sirolimus^®^ and Everolimus^®^. It forms a complex with FK506 binding protein 12 (FKBP12), which allows inhibition of mTOR. The mTOR pathway can be activated in several ways, among them the variation in the ATP:AMP ratio via AMP-activated protein kinase (AMPK); by insulin and insulin-like growth factor 1 (IGF-1); by amino acids; via Rag GTPases; and via the activation by Wnt of the glycogen-synthase kinase 3 (GSK3) pathway. Immune system receptors, such as TCR, BCR, cytokine receptors and TLRs, are also able to activate mTOR (Weichhart and Säemann, 2009). All of proteins recruit kinases, such as phosphoinositide 3-kinase (PI3K), that, in turn, phosphorylate phosphatidylinositol 4,5-bisphosphate (PIP2) and phosphatidylinositol 3,4,5-triphosphate (PIP3). This process activates the serine-threonine kinase Akt, which phosphorylates tuberous sclerosis complex 2 (TSC-2) and decreases the inhibition of Rheb, which activates mTOR (Huang and Manning, 2009). mTOR participates in two complexes, the first of which is known as mTOR complex 1 (mTORC1) and forms part of the regulatory protein associated with mTOR (Raptor) (Kim and Sabatini, 2004; Zhou and Huang, 2010). A second complex, known as mTORC2, is also composed of mTOR, DEPTOR and Rictor (Sarbassov et al., 2004; Zhou and Huang, 2010). Although mTOR activation controls the cellular metabolism of immune cells, its role in regenerative and fibrotic processes is unknown thus far.

In recent years, new discoveries have contributed substantially to improved understanding of the mechanisms involved in fibrosis and has highlighted the factors related to cellular metabolism (Grande et al., 2015; Lovisa et al., 2015). Kang et al. demonstrated that inhibition of fatty acid metabolism in renal tubular cells by etomoxir was able to induce apoptosis, fibrosis and the depletion of ATP, which was reflected in greater expression of mesenchymal marker genes, such as ACTA2 (which encodes α-SMA), vimentin, and genes encoding fibrillar collagens. In addition, the treatment of tubular epithelial cells with TGF-β1 leads to reduced expression of important factors involved in the regulation of the metabolism of fatty acids, such as carnitine palmitoyltransferase-1 (CPT1), PPAR-α and PGC1-α (Kang et al., 2015). Moreover, it has been found that, in the CNS, the mTOR pathway plays a critical role in regulating the regenerative capacity of neurons, in contrast to its role in the peripheral nervous system (Huang et al., 2017). In this sense, mTOR maintains the axonal growth state in the contexts of sepsis (Guo et al., 2017) and other inflammatory stimulation (Leibinger et al., 2012).

A correlation between mitochondrial energy metabolism and liver regeneration twenty-4 h post partial hepatectomy was also identified. During the early phase of liver regeneration, there was a decrease in the respiratory control index and in the amounts of the two subunits of ATP synthase in mitochondria, which led to decreased oxidative phosphorylation. After this initial phase, the levels of these mitochondrial subunits of ATP synthase increased and oxidative phosphorylation was recovered, suggesting that the regeneration capability of rat liver is correlated with the efficiency of mitochondrial energy metabolism (Guerrieri et al., 1995).

Studies in zebrafish regarding metabolic processes during regeneration are still lacking. A proteomic profile of the zebrafish caudal fin in its native state was generated, and a total of 417 proteins were identified to be specific to zebrafish fin tissue and were demonstrated to be involved in various biological activities related to development, apoptosis, signaling and metabolic processes (Singh et al., 2011). Furthermore, another study of caudal fin regeneration in adult zebrafish has uncovered complex overlapping expression patterns comprising hundreds of molecules that are involved in diverse cellular functions, including amino acid and lipid metabolism (Rabinowitz et al., 2017). In addition, a study compared the proteomic profile of the retina upon regeneration and during injury in an experimental degeneration model. The cytoskeleton and membrane transport proteins were altered during regeneration, with the highest fold-change upregulation observed for β-2 tubulin A, histone H2B and brain type fatty acid binding protein. In addition, via gene ontology analysis, reduced metabolic processing and increased fibrin clot formation during degeneration were observed when compared to that in the normal retina (Eastlake et al., 2017). However, further investigations of the energy metabolism of immune cells during zebrafish regeneration are needed.

## Mitochondria and Redox State

Mitochondria are organelles present in nucleated cells that act in a dynamic way and are fundamental to the regulation of energy metabolism processes. Mitochondria are primordial in terms of several cellular processes, such as energy generation (ATP), calcium homeostasis, steroid biosynthesis, ROS production, apoptosis and cell cycle regulation (Akhmedov and Marín-García, 2015). They are sensitive to some changes and respond to stimuli such as hormones, oxygen, and nutrients. Responses to rapid and sensitive metabolic changes are provided by redox cellular targets (Handy and Loscalzo, 2012).

Reactive oxygen species, in particular, are highly influential modulators of cellular function. Until recently, ROS were studied because of their deleterious effects on proteins, lipids and DNA, which can culminate in cell death and injury. They are currently emphasized for their role in modulating processes through redox-dependent signaling when present at low concentrations (Rigoulet et al., 2011). When in excess, ROS disrupt cellular homeostasis and promote damage to macromolecules. Therefore, it is known that a balance is needed between the production and elimination of ROS (Handy and Loscalzo, 2012).

The electron transport chain (ETC) and enzymatic synthesis are recognized as sources of ROS. The major source of these species is the mitochondria, especially the mononucleotide flavin (FMN) site of complex I and the Q cycle of ETC complex III (Nickel et al., 2014). It has been suggested that, of the total oxygen consumed, 2 to 5% is destined for the generation of superoxide, of which 70 to 80% originates from the leakage of electrons from the type III complex. It is also worth mentioning that the generation of these radicals varies according to the cell type and the respiratory state of the cell. The mechanism of superoxide formation involves the incomplete reduction of molecular oxygen due to the leakage of electrons from the chain during the process of normal cellular respiration (Rigoulet et al., 2011; Handy and Loscalzo, 2012).

**FIGURE 2:**
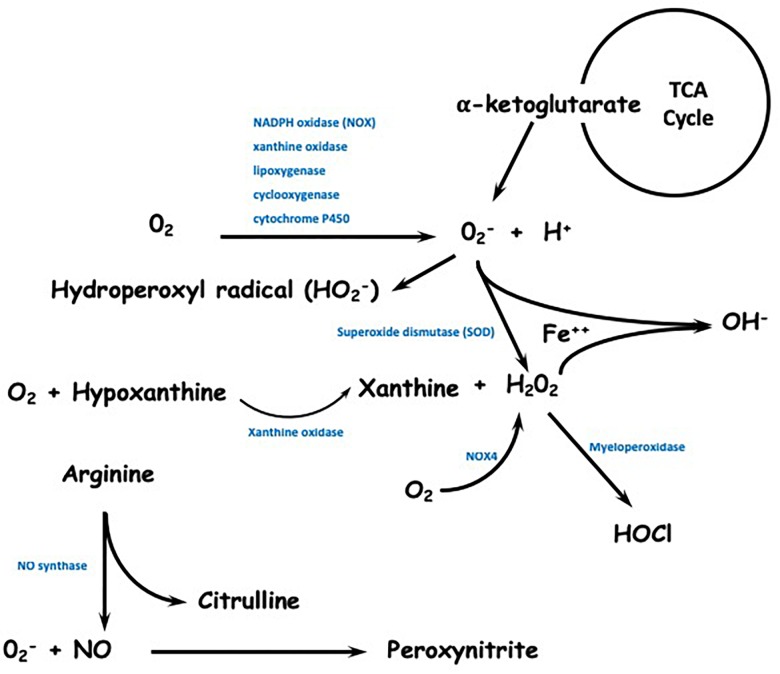
Schematic representation of reactive species formation. Reactive species can be synthesized through the reduction of molecular oxygen and the formation of superoxide ion radicals by the enzymes NADPH oxidase (mainly NOX2), xanthine oxidase, lipoxygenase, cyclooxygenase, and cytochrome P450. The α-ketoglutarate dehydrogenase complex, which is present in the citric acid cycle, can also be a source of mitochondrial superoxide generation. Superoxide can become protonated to generate hydroperoxyl and can be reduced to hydrogen peroxide spontaneously or more rapidly, through the action of superoxide dismutase enzymes (SOD). The synthesis of hydrogen peroxide can also be performed by xanthine oxidase enzymes and NOX4. Hydrogen peroxide is able to diffuse through biological membranes and has a longer half-life. Reactive nitrogen species (RNS) are mainly formed through the action of NO synthase.

Reactive species can also be synthesized through the reduction of molecular oxygen and the formation of superoxide ion radicals by the enzymes xanthine oxidase, cytochrome P450 (Hrycay and Bandiera, 2015), lipoxygenase, and cyclooxygenase (Kontos et al., 1985) and by isoforms of NADPH oxidase, mainly NOX2 (Holmström and Finkel, 2014). In addition to these enzymes, the α-ketoglutarate dehydrogenase complex, which is present in the citric acid cycle, can also be a source of mitochondrial superoxide generation (Phaniendra et al., 2015). Superoxide can combine with nitric oxide to form the reactive nitrogen species (RNS) peroxynitrite. Superoxide can also be protonated to generate hydroperoxyl, which is a radical that promotes lipid peroxidation followed by the oxidative modification of proteins, low molecular weight thiols and lipid membranes (Adams et al., 2015). Finally, superoxide can be reduced to hydrogen peroxide (H_2_O_2_) spontaneously or, more rapidly, through the action of superoxide dismutase enzymes (SODs). The synthesis of hydrogen peroxide can also be performed by xanthine oxidase enzymes and NADPH oxidase 4 (NOX4) by reduction of two flavin-dependent electrons (Brown and Griendling, 2009; Kelley et al., 2010). Hydrogen peroxide, which is a neutral and less reactive species compared to superoxide, is able to diffuse through biological membranes and has a longer half-life. Because of these characteristics, this molecule becomes extremely relevant in redox signaling but in excess, it contributes to cellular oxidative stress (Handy and Loscalzo, 2012). A schematic representation of ROS and RNS is shown in Figure 2.

As the main source of oxidants in cells, mitochondria are essential to the basal production of these reactive species and the maintenance of normal cellular function through regulation of the redox state (Holmström and Finkel, 2014). It is known that some protein subgroups have heightened sensitivity to the oxidation of the thiol group, which is present in complexes I, II and IV of the ETC (Clementi et al., 1998; Handy and Loscalzo, 2012). In mitochondria, the posttranslational modifications of some subunits, such as complex I, may influence the generation of superoxide (Grivennikova and Vinogradov, 2006). Specifically, thiol oxidation of complex I may promote radical production, while S-nitrosation suppresses complex activity by reducing the leakage of electrons through this complex. Other modulations to control the leakage of electrons can be performed by sirtuins located in the mitochondria, which regulate the acetylation of complexes I and II (Handy and Loscalzo, 2012). A study has been conducted to evaluate the overexpression of glutathione peroxidase 1 (GPx-1), which led to a decrease in the synthesis of cellular ROS and resulted in a reduction in disulfide bonds and alterations in mitochondrial function, such as decreased production of ATP (Birben et al., 2012; Handy et al., 2009). Other modifications of the antioxidant systems of organisms can cause several changes in cellular and mitochondrial metabolism and promote the cellular modifications detailed in Section “Antioxidant Systems.”

### Redox State and Regeneration

The role of H_2_O_2_, a chemically stable ROS that can diffuse rapidly (Bienert et al., 2006), during tail fin regeneration in zebrafish larvae has been revealed using a genetically encoded H_2_O_2_ sensor. This reporter has shown a sustained rise in H_2_O_2_ concentration at the wound margin that starts approximately 3 min after injury and peaks after 20 min and that extends 100–200 μm into the tail epithelium and presents a decreasing concentration gradient. Through genetic and pharmacological inhibition of dual oxidase (Duox), it has been demonstrated that its activity is decisive for leukocyte recruitment to the wound because Duox is responsible for the generation of a tissue-scale H_2_O_2_ gradient after tail fin wounding in zebrafish (Niethammer et al., 2009).

Following adult fin amputation in zebrafish, ROS production is tightly regulated spatiotemporally for at least 24 h, and ROS signaling triggers two different pathways. One of these pathways is responsible for c-Jun N-terminal kinase (JNK) activation and the other is responsible for apoptosis. Both pathways are involved in the Wnt, stromal cell-derived factor 1 (SDF1) and IGF pathways, while the latter impacts progenitor marker expression as well. These two events, JNK activation and apoptosis, are fundamental for the compensatory proliferation of epidermal cells, are essential to blastema formation and are consequently relevant for proper regeneration (Gauron et al., 2013). In a previous work (Niethammer et al., 2009), the effect of NOX inhibition was assessed using VAS2870 or DPI, which resulted in the greatly reduced size of the regenerated fin at 72 h postamputation (hpa) when NOX was inhibited from the amputation time until 72 h later. When inhibition occurred from 12 to 24 hpa, the resulting regeneration was similar, suggesting that during this time frame, NOX participates in an early signaling pathway involved in blastema formation (Gauron et al., 2013).

Similarly, it has been demonstrated that H_2_O_2_ plays a crucial role in cardiac regeneration in adult zebrafish (Han et al., 2014). Through live imaging of hearts, hydrogen peroxide production was seen in the epicardium and adjacent compact myocardium at the resection site. By using the Duox inhibitors DPI or apocynin or scavenging H_2_O_2_ by catalase overexpression, impaired cardiac regeneration was observed, while exogenous H_2_O_2_ rescued the inhibitory effects of DPI, which suggested that hydrogen peroxide is an essential signal in this biological process (Han et al., 2014). In addition to being important for the mediation of leukocyte recruitment to wounds through the Src family kinase (SFK) redox sensor Lyn (Yoo et al., 2011), H_2_O_2_ promotes heart regeneration by initiating MAP kinase signaling through a derepression mechanism involving Dusp6, which is a redox-sensitive phosphatase. Dusp6 is destabilized by H_2_O_2,_ resulting in increased phosphorylation of extracellular signal-regulated kinase 1/2 (Erk1/2). When Dusp6 is inhibited, pro-regenerative effects are achieved. In contrast, its overexpression in a transgenic manner impaired cardiac regeneration (Han et al., 2014).

As a key mechanism involved in the local resolution of inflammation, the reverse migration of neutrophils, which is the process by which these cells emigrate from inflamed tissues (Mathias et al., 2006; Woodfin et al., 2011; Starnes and Huttenlocher, 2012; Robertson et al., 2014), has been addressed by recent studies. An investigation of fin transection using a double transgenic zebrafish, Tg(*mpx:DsRed*) × Tg(*mpeg1:Dendra2*), has demonstrated that redox-SFK signaling via p22phox and Yes-related kinase (Yrk) is crucial for the recruitment of macrophages to wounds and for the successive reverse migration of neutrophils (Tauzin et al., 2014). In addition to the role of Duox in leukocyte recruitment, it has been found that macrophages may sustain their directed migration into injured tissue by producing their own source of ROS, as demonstrated by the use of *cyba* morpholino to inhibit p22phox, a subunit of Nox2 that is expressed in both neutrophils and macrophages, which partially impaired macrophage recruitment to the wound (Tauzin et al., 2014). Additionally, it was shown that the presence of macrophages in wounds does not impact initial neutrophil recruitment but is necessary for the resolution of neutrophil infiltration at sites of tissue damage (Tauzin et al., 2014). Furthermore, Yrk, which is expressed exclusively in macrophages, has been investigated for its possible role in chemotaxis. Using two different morpholinos, endogenous Yrk was depleted, which impaired early and late recruitment of macrophages to wounds. Based on these results concerning phagocyte wound chemotaxis, it was possible to predict that ROS-SFK differentially regulates late neutrophil and macrophage wound recruitment. Finally, it has been indicated that macrophages are required for neutrophil reverse migration and the subsequent resolution of neutrophil-mediated inflammation at wounds, suggesting that neutrophils move away from the wound through contact-mediated guidance from macrophages (Tauzin et al., 2014).

### Antioxidant Systems

As discussed earlier, cells need to maintain a balance between the production and removal of ROS. Thus, there are a number of antioxidant processes. Despite extra mitochondrial synthesis, mitochondrial function is controlled by the redox signaling processes that occur throughout the cell and also, possibly, by the redox state of the extracellular space (Nickel et al., 2014). Manganese-dependent superoxide dismutase enzyme (MnSOD), an antioxidant enzyme, is present in the mitochondrial matrix (Miriyala et al., 2012), and it is responsible for reducing superoxide in hydrogen peroxide. Glutathione peroxidase 1 (GPx-1), another antioxidant enzyme, is also located in the mitochondrial matrix and uses reduced glutathione (GSH) as an obligate cosubstrate in the reduction of hydrogen peroxide to water (Lubos et al., 2011). Similarly, peroxiredoxins (Prx3 and Prx5) act by reducing peroxide derived from thioredoxin 2 (Trx2). In addition, they are complementary to glutathione reductase (GR) and thioredoxin reductase (TrxR), which reduce the previously oxidized glutathione and thioredoxin molecules via free radical reduction processes (Fujii and Ikeda, 2002). In the mitochondrial intermembrane space, it has been proposed that copper- and zinc-dependent superoxide dismutase (Cu/Zn SOD) can reduce the superoxide synthesized by ETC complex III. Additionally, in the intermembrane space and in the cytosol, GPx-1 and Prx perform the same function as radicals produced by other sources (Esposito et al., 2000; Handy and Loscalzo, 2012).

Glutathione peroxidases are found in two different redox states: either in the reduced form (GSH) or the oxidized form [glutathione disulfide (GSSG)]. Glutathione peroxidases are composed of selenocysteine, with the exception of GPx-5. Some of these enzymes, such as GPx-1, rely on GSH as a cofactor to reduce hydrogen peroxide and lipid hydroperoxides, as discussed above (Espinosa-Diez et al., 2015). Others rely on thioredoxin, which is also used as a reducing source for peroxiredoxins. Thus, NADPH storage is required for the recycling of GSH and Trx, since these are oxidized to GSSG and oxTrx and require the action of the enzymes glutathione reductase and thioredoxin reductase, respectively, both of which use NADPH as a cofactor during enzymatic processing (Toledano et al., 2013). GPx-1 is localized to the mitochondria, cytosol and peroxisomes, and it modulates mitochondrial function. An increase in GPx-1 expression, therefore, leads to decreases in the mitochondrial electrochemical potential, ROS generation and ATP production (Lubos et al., 2011; Handy and Loscalzo, 2012).

A member of another group of antioxidant enzymes found abundantly in organisms, extracellular superoxide dismutase (ecSOD), is a glycoprotein that is secreted and can associate with cell surface glycosaminoglycans to protect against the effects of superoxide outside of the cell (Ota et al., 2016). Thus, this enzyme plays a role in preserving vascular function, since it is able to attenuate the inactivation of NO by superoxide, which maintains NO-mediated vasodilation responses in endothelium-dependent smooth muscle cells as well as limits oxidative damage caused by the formation of peroxynitrite (Qin et al., 2008). It is also known that changes in the extracellular redox potential are capable of promoting changes in the intracellular redox balance, which affects mitochondrial ROS production. Thus, it can be assumed that changes in the levels of extracellular antioxidants may influence cellular function, since these changes can influence the redox status of the cell (Imhoff and Hansen, 2009).

Catalase, a tetrameric enzyme containing heme, is involved in the reduction of hydrogen peroxide to water in the intracellular space and more specifically, in the peroxisomes (Zamocky et al., 2008). The functioning of catalase does not depend on NADPH; however, the binding of NADPH to each of the four catalase monomers prevents and reverts the conversion of this enzyme to an inactive state via hydrogen peroxide through the supply of electrons to NADPH. Moreover, this binding optimizes the action of catalase and stabilizes it in the tetrameric form (Kirkman et al., 1999). As catalase is found only within a restricted site (peroxisomes), it is not the main mechanism that modulates oxidative responses in an organism (Scibior and Czeczot, 2006). Despite this, studies suggest that catalase plays a central role in some tissues, such as in the heart and liver of rats and, combined with glutathione peroxidase, in erythrocytes (Gaetani et al., 1989; Kirkman et al., 1999).

As previously mentioned, NADPH is of great relevance to the maintenance of the activity of antioxidant systems, especially those systems that reduce hydrogen peroxide and its derivatives. An enzyme that participates in the pentose-phosphate metabolic pathway, glucose 6-phosphate dehydrogenase (G6PD), is essential for the synthesis and maintenance of NADPH through its catalysis of the reduction of NADP^+^ to NADPH, which is a process that is important in maintaining the redox balance in cells (Stanton, 2012). Deficiency in G6PD is associated with oxidative stress, whereas its overexpression protects cells from the accumulation of ROS. In humans, the deficient or low activity variant of G6PD is very common (Luzzatto et al., 2016) and is especially abundant in regions where malaria is endemic, which can be explained by the fact that this variant confers protection against severe forms of the disease by decreasing erythrocyte removal that results from the reduction of oxidative lesions. In addition, deficiency in G6PD may increase susceptibility to diseases such as diabetes and hypertension, which are also a result of redox imbalances and oxidative stress (Cappadoro et al., 1998).

In addition to G6PD, there are other pathways related to NADPH biosynthesis. The nicotinamide nucleotide transhydrogenase (NNT) pathway, which is modulated by the electrochemical gradient in mitochondria, is also able to regenerate NADPH from NADP^+^ by using NADH synthesized in the citric acid cycle (Lopert and Patel, 2014). Thus, deficiencies in mitochondrial NADH or alterations in the membrane potential may be responsible for the decreased regeneration of NADPH, which will contribute to oxidative stress (Stein and Imai, 2012). Moreover, the NADPH reserve can be recovered by some dehydrogenases that are present in mitochondria, such as isocitrate dehydrogenase (ICDH) and malate dehydrogenase (MDH) (Rydström, 2006).

### ROS and Mitochondrial Biogenesis

Mitochondria are seen as dynamic organelles that are constantly subjected to fusions and fissions based on cellular metabolic demands (Benard et al., 2007). The mitochondrial genomic content and the number of mitochondria varies from cell to cell, and these characteristics are related to responses to different growth factors and other nutrients capable of promoting the synthesis or biogenesis of new mitochondria and the removal of damaged mitochondria through mitophagy (Cherry and Piantadosi, 2015). Mitochondrial biogenesis is a complex process involving a number of genes and regulators as well as other factors. For biogenesis to occur, the coordinated expression of genes in the nuclear and mitochondrial genomes is required. Specifically, 13 subunits in oxidative phosphorylation complexes are encoded by mtDNA (Kirby and Thorburn, 2008). The other components, including other essential proteins, are derived from nuclear DNA. Transcription factors such as nuclear respiration factor 1 and nuclear respiration factor 2 (NRF1 and NRF2) participate in the transcriptional regulation of oxidative phosphorylation genes and antioxidant genes as well as those involved in mtDNA replication and transcription (Jornayvaz and Shulman, 2010). NRF1 and NRF2 are activated in part via the peroxisome-proliferator-activated receptor γ coactivator-1α (PGC-1α) and peroxisome-proliferator-activated receptor γ coactivator-1 β (PGC-1β) protein receptors (Puigserver et al., 1998; Wu et al., 1999).

In some models, acute stimulation with exogenous oxidants was able to activate mitochondrial biogenesis, as observed by the increase in mitochondrial mass and mtDNA as well as the expression of biogenic regulators such as PGC-1α, NRF1 and transcription factor mitochondrial A (TFAM) (St-Pierre et al., 2003; Kukidome et al., 2006). However, in other models, it was demonstrated that oxidant-induced acute injuries initially led to mitochondrial dysfunction that was characterized by posterior mitochondrial repair that resulted from PGC-1α regulation. Mitochondrial biogenesis mediated by PGC-1α may promote the substitution of dysfunctional mitochondria, which are subject to increased electron leak, with more efficient mitochondria, effectively reducing the production of mitochondrial ROS. In addition, PGC-1α proteins can also decrease ROS synthesis once ROS induce the synthesis of antioxidant proteins, such as catalase, SOD2, GPx-1, Trx2, decoupling protein 2 (UCP2), and Prx3 (Valle et al., 2005; St-Pierre et al., 2006).

### Oxidative Stress and Damage in Zebrafish

By defining cellular processes in aquatic organisms, it is possible to verify the induction of oxidative stress by environmental pollutants. Some toxic substances stimulate a series of reactions in aquatic organisms that, through the synthesis of free radicals and ROS production, can cause damage to tissues and cellular components (Lushchak, 2011). Similarly, but in a more tenuous way, we also observed the production of ROS and protein oxidation during regeneration in animals (Gauron et al., 2013; Love et al., 2013; Wu et al., 2017). A recent study showed that ROS released when zebrafish were wounded was responsible for the repositioning of notochord cells to the damaged site and stimulated these cells to secrete the Hedgehog ligands that are essential for regeneration (Romero et al., 2018).

Molecular biomarkers are used to evaluate oxidative damage in biomolecules or to evaluate oxidative stress (Kumari et al., 2014). As described earlier, the antioxidant defense system can be separated into enzymatic antioxidants, such as catalase, superoxide dismutase, and glutathione peroxidase, and non-enzymatic antioxidants, such as glutathione, ascorbate, and vitamin E. Biomarkers are used to analyze lipid peroxidation, DNA damage, and protein oxidation and to determine the level of activity of antioxidant enzymes (Valavanidis et al., 2006).

Aquatic organisms are more sensitive to toxicity than terrestrial organisms, including mammals, and thus, they may be useful models for evaluating the more subtle effects of oxidative stress (Valavanidis et al., 2006). One of the most heavily investigated processes is lipid peroxidation, which is measured in most techniques by the levels of secondary oxidized products due to the difficulty of the direct analysis of endogenous peroxidation (Devasagayam et al., 2003). The most commonly used test measures the formation of malondialdehyde (MDA). Another widely studied process is the carbonylation of proteins; in particular, the oxidation products of the amino acids phenylalanine and tyrosine are commonly measured (Halliwell and Whiteman, 2004). The oxidation of the latter leads to the formation of dityrosine, which is an important biomarker of oxidative stress (Valavanidis et al., 2006).

Several markers of oxidative stress have been investigated in fish gills, kidneys and liver. Lipid peroxidation, specifically, was measured in the muscle and liver of fish to reveal the degree of pollution caused by an oil refinery (Valavanidis et al., 2006). In addition, regarding cellular antioxidants, fish have a distinct response to exposure to pollutants with oxidative potential. When compared to other vertebrate organisms, fish exhibit lower baseline superoxide dismutase and catalase activity and higher glutathione peroxidase activity (Rau et al., 2004). Additionally, during ROS-induced inflammation, the concentration of antioxidant enzymes is increased; however, when fish are subjected to high concentrations of pollutants, the activity of this defense system is reduced (Valavanidis et al., 2006).

There are a variety of antioxidant enzymes with significant biological activity that protect organisms against the toxic effects of ROS (Dröge, 2002). The expression of genes encoding antioxidant enzymes is regulated by a central factor in zebrafish, NRF2 (Ma, 2013). It has been suggested that this factor, under normal physiological conditions, binds to Kelch-like ECH-associated protein 1 (Keap1) and is targeted for proteasomal degradation. However, Nrf2-Keap1 dissociation leads to its nuclear translocation, which allows NRF2 to dimerize with musculoaponeurotic fibrosarcoma oncogene (Maf), and the binding of this heterodimer to the antioxidant response element (AER) enables the transcriptional activation of genes such as glutathione S-transferase P1 (*GSTp1*) in zebrafish. GSTp1 therefore plays an important role in cellular protection against oxidative stress and toxic foreign chemicals (Jiang et al., 2014).

### Mitochondria and Regeneration in Zebrafish

The contribution of mitochondria to tissue regeneration has been widely studied. Presently, it is known that the maintenance of these organelles through the autophagic process favors the remodeling and functional regeneration of skeletal muscle in mice (Nichenko et al., 2016). Autophagy is also required during caudal fin regeneration in zebrafish. The genetic and pharmacological inhibition of autophagy, which has been described as a process of autodegradation mediated by lysosomes that occurs in eukaryotes, leads to deficient tail regeneration in *D. rerio* and demonstrates that this process is necessary for autophagy-induced tissue renewal (Varga et al., 2014).

Mitochondria are closely linked to cell survival. There are several pathways by which apoptosis can be induced, and it is of great relevance to discover which of these pathways are related to the regeneration process (Elmore, 2007). The expression of closely related genes has revealed that there are several apoptotic pathways that regulate the biological process of liver regeneration (Xing et al., 2016). In addition to the previously discussed mechanisms, the mitochondria have other important functions, such as providing energy and producing metabolites required for cell proliferation to occur. In studies performed using mice, it was shown that knockout of the TOP1mt gene led to a deficiency in liver regeneration and a reduction in reduced glutathione (GSH), which reflected the increased production of ROS. Thus, mitochondrial topoisomerase is required for normal homeostasis and for the expansion of mitochondrial DNA during hepatocyte proliferation (Khiati et al., 2015).

Liver regeneration is stimulated by several growth factors, and augmenter of liver regeneration (ALR) plays a central role in this process. ALR is also known as hematopoietin and is located in the nucleus, cytoplasm and intermembrane space of the mitochondria. It has been reported to be involved in oxidative phosphorylation, mitochondrial maintenance and biogenesis, autophagy regulation and cell proliferation (Gandhi, 2012; Nalesnik et al., 2017). ALR participates in reversing the inhibition of the cell cycle and maintaining the stock of stem cells. In addition, ALR is known to be important during the developmental stages of vertebrates. In a study that used zebrafish as a model, the overexpression of ALR led to abnormal liver growth. However, the knockdown of ALR caused the inhibition of the development of this organ (Li et al., 2012). In another study, the expression of ALR was linked to the expression of TNF-α and IL-6 (Vodovotz et al., 2013). TNF-α and IL-6 were also involved in improved regeneration on days 1 and 2, respectively, and these two molecules were expressed at high levels on day 4; the maximum level of ALR was also observed on day 4. It has been shown that this protein is increased when the liver is injured; thus, because it is a damage-sensitive molecule, ALR can be used as an indicator of hepatic stress (Vodovotz et al., 2013; Gupta and Venugopal, 2018).

A biological cellular mechanism involved in axon regeneration has also been demonstrated. This process is regulated by DLK-1, which stimulates the translocation of mitochondria to the injured region to increase the mitochondrial mean density (Mar et al., 2014; Patrón and Zinsmaier, 2016). In the same study, it was also demonstrated that alterations in both mitochondrial density and respiratory chain functioning promoted modification of the regenerative effect. Therefore, axons that do not promote an increase in mitochondrial density have poor regeneration (Han et al., 2016). Finally, it has been demonstrated that some genes participate in epigenetic regulation of tail fin regeneration in zebrafish. Among them, Dnmt3aa, Dnmt3ab and Dnmt4, which are genes that have orthologs in mammals (Takayama et al., 2014), are responsible for DNA methylation patterns during development (Seritrakul and Gross, 2014).

## Concluding Remarks

The understanding of the processes of tissue regeneration is of paramount importance for improved understanding of several diseases. *D. rerio*, which is a species with high regenerative capacity in its tail and in more complex organs, has been increasingly used in research involving regeneration. Macrophages and neutrophils play a very important role in this process, specifically in regulating pathways related to redox balance and oxidative processes. In recent years, several studies in the areas of pathology and immunology have attempted to pinpoint the functions of these cell types in harmful, regenerative and fibrotic processes. However, there is still much to be discovered about the metabolic profiles of neutrophils and infiltrating macrophages during the regenerative process in zebrafish. There is even more to be discovered in terms of the translation of this knowledge from the bench to the bedside, which will allow us to apply knowledge learned from zebrafish models to clinical practice (Agrahari et al., 2019; Chan and Viswanathan, 2019) during wound lesion treatment and the recovery of fibrotic tissues through clinical modulation of immune cells.

## Author Contributions

LCP and TB wrote the article. NC revised it.

## Conflict of Interest Statement

The authors declare that the research was conducted in the absence of any commercial or financial relationships that could be construed as a potential conflict of interest.
